# Enhancing Terminal Deoxynucleotidyl Transferase Activity on Substrates with 3′ Terminal Structures for Enzymatic De Novo DNA Synthesis

**DOI:** 10.3390/genes11010102

**Published:** 2020-01-16

**Authors:** Sebastian Barthel, Sebastian Palluk, Nathan J. Hillson, Jay D. Keasling, Daniel H. Arlow

**Affiliations:** 1 Joint BioEnergy Institute, Emeryville, CA 94608, USA; uni.sbarthel@gmail.com (S.B.); sebastian.palluk@gmail.com (S.P.); njhillson@lbl.gov (N.J.H.); jdkeasling@lbl.gov (J.D.K.); 2 Biological Systems and Engineering Division, Lawrence Berkeley National Lab, Berkeley, CA 94720, USA; 3 Department of Biology, Technische Universität Darmstadt, 64287 Darmstadt, Germany; 4 DOE Joint Genome Institute, Walnut Creek, CA 94598, USA; 5 Institute for Quantitative Biosciences, UC Berkeley, Berkeley, CA 94720, USA; 6 Department of Chemical and Biomolecular Engineering, UC Berkeley, Berkeley, CA 94720, USA; 7 Department of Bioengineering UC Berkeley, Berkeley, CA 94720, USA; 8 Novo Nordisk Foundation Center for Biosustainability, Technical University of Denmark, 2970 Hørsholm, Denmark; 9 Center for Synthetic Biochemistry, Institute for Synthetic Biology, Shenzhen Institutes for Advanced Technologies, Shenzhen 518055, China; 10 Biophysics Graduate Group, UC Berkeley, Berkeley, CA 94720, USA

**Keywords:** enzymatic DNA synthesis, terminal deoxynucleotidyl transferase, TdT, secondary structures, oligonucleotide synthesis, template-independent polymerase, DNA data storage, thermostability engineering, polymerase cofactors

## Abstract

Enzymatic oligonucleotide synthesis methods based on the template-independent polymerase terminal deoxynucleotidyl transferase (TdT) promise to enable the de novo synthesis of long oligonucleotides under mild, aqueous conditions. Intermediates with a 3′ terminal structure (hairpins) will inevitably arise during synthesis, but TdT has poor activity on these structured substrates, limiting its usefulness for oligonucleotide synthesis. Here, we described two parallel efforts to improve the activity of TdT on hairpins: (1) optimization of the concentrations of the divalent cation cofactors and (2) engineering TdT for enhanced thermostability, enabling reactions at elevated temperatures. By combining both of these improvements, we obtained a ~10-fold increase in the elongation rate of a guanine-cytosine hairpin.

## 1. Introduction

Oligonucleotide synthesis is essential for modern biological research and promises to enable new “digital biology” applications, such as DNA-based data storage and computation [1]. Currently there is only one method available for oligonucleotide synthesis: The nucleoside phosphoramidite method [2]. However, this method has several key disadvantages for emerging DNA applications, including that (1) it is limited to the direct synthesis of high-quality ~250 mers [2] and (2) it strictly uses anhydrous solvents and produces a toxic and flammable waste stream, making it burdensome to operate in new settings, such as a data center or a spacecraft [3].

Recently, a new class of oligonucleotide synthesis methods based on the template-independent polymerase terminal deoxynucleotidyl transferase (TdT) have been described [4,5]. Enzymatic procedures promise improved synthesis yields and, thus, increased oligonucleotide lengths, with synthesis occurring in mild, aqueous conditions. TdT is a unique member of the DNA polymerase X family that indiscriminately adds deoxynucleoside triphosphates (dNTPs) to the 3′ end of a single-stranded DNA (ssDNA) primer. For the synthesis of defined sequences using TdT, a “reversible termination” mechanism is required to ensure that a single nucleotide is added to each growing chain in a given reaction, so that, after the reaction is completed, the chains can be unblocked to enable elongation with the next desired nucleotide. One approach uses nucleotides with a removable blocking group on the 3′ OH of the incoming dNTP [6]. However, 3′ O-modified dNTPs are not readily incorporated by TdT, so engineering of the catalytic site is required. We recently developed a novel method that does not require a 3′ O-modified dNTP and instead achieves reversible termination by covalently tethering one dNTP to each TdT molecule via a cleavable linker [5]. An alternative method for synthesizing DNA specifically for DNA-based data storage relies on homopolymeric runs and, thus, avoids the need for reversible termination [7,8], but cannot produce the precise DNA sequences required for biological applications.

TdT-based oligonucleotide synthesis takes place under aqueous conditions in which the growing ssDNA molecules will inevitably form secondary structures once they reach a certain length. Since TdT has poor activity on substrates with a secondary structure creating a recessed 3′ OH [9], we were concerned that 3′ terminal secondary structures that arise during synthesis could lead to poor coupling yields and, thus, deletions in the synthesized oligonucleotide. Here, we sought to investigate the impact of 3′ terminal structures on TdT-mediated elongation reactions and to identify potential mitigations for inhibition. In particular, we observed dramatically reduced elongation rates of guanine-cytosine (GC) hairpins and were able to partially restore activity by simultaneously lowering the divalent ion concentration and raising the reaction temperature.

## 2. Materials and Methods 

Several methods were previously described in Palluk et al. [5] and are briefly summarized below, including the DNA construction of the MTdTwt-encoding plasmid, analysis via capillary electrophoresis, and the generation of polymerase-nucleotide conjugates.

### 2.1. DNA Construction 

Within this study, we used the polymerase domain of wild-type TdT from *Mus musculus* (NCBI Accession number: NP_001036693.1) fused to an N-terminal maltose-binding protein (MBP) and a 10x His-tag (MTdTwt, Joint BioEnergy Institute, JBEI, Public Registry: JPUB_013230). To engineer TdT variants with enhanced thermostability (MTdT-evo and MTdTc302-evo, JBEI Public Registry: JPUB_013230 and JPUB_013228), we used the FireProt algorithm [10] via its web interface [11]. Calculations were based on a the crystal structure of TdT PDB ID: 4I27 [12] and run with default settings. All suggested mutations from FireProt’s evolution-based approach were accepted except for the Leu398Met mutation, since Leu398 is known to be important for the functionality of the protein [12]. The FireProt predictions based on 4I27 were also used to design MTdTc302-evo, which additionally contained the same cysteine mutations as MTdTc302 in Palluk et al. (Cys188Ala, Cys216Ser, Cys378Ala, Cys438Ser, PDB ID 4I27 numbering [5]) and can be used for the preparation of polymerase-nucleotide conjugates. The genes encoding MTdT-evo and MTdTc302-evo were codon-optimized using the manufacture’s codon optimization tool, ordered from Integrated DNA Technologies (IDT; Coralville, IA, USA), and then inserted into a pET19b vector via isothermal assembly. For further information, including a list of all plasmids and expression strains with the respective JBEI Public registry accession number (Table S1) and all protein sequences, see Note S1. All strains were deposited in the JBEI strain archive and are available upon request (https://public-registry.jbei.org/folders/432). The electropherogram data and custom analysis software are available from the authors upon request.

### 2.2. Protein Expression and Purification of MBP-Fused TdT

*Escherichia coli* BL21 (DE3) harboring pET19b-MTdTwt, pET19b-MTdT-evo, or pET19b-MTdTc302-evo were grown in LB-Miller medium with 100 μg/mL carbenicillin and 1% (*w*/*v*) D-glucose at 200 rpm shaking throughout the process. Starting cultures were grown overnight at 37 °C and used to inoculate expression cultures to an OD_600_ of 0.1. Expression cultures were grown to an OD_600_ of 0.8 at 37 °C and then transferred to an incubator at 15 °C for a 30-min incubation without shaking. Afterwards, protein expression was induced with 1 mM isopropyl β-D-1-thiogalactopyranoside (IPTG) and cells were grown overnight at 15 °C with shaking. Cells were harvested by centrifugation (5000× *g*, 15 min, 4 °C). Cell pellets were stored at −80 °C if not immediately used for protein purification. All protein purification steps were performed at 4 °C. First, gravity columns were packed with 2 mL Ni-NTA agarose resin (Qiagen; Germantown, MD, USA) and equilibrated with 10 mL Buffer A (20 mM Tris-HCl, 0.5 M NaCl, pH 8.3) plus 5 mM imidazole. Cells were lysed in Buffer A plus 5 mM imidazole using a QSonica Q700 sonicator, cell debris were removed by centrifugation at 15,000× *g* for 20 min, and the supernatant was loaded onto gravity columns. The columns were washed twice with 10 mL Buffer A plus 40 mM imidazole and proteins were eluted with 4 mL Buffer A plus 500 mM imidazole. Proteins were buffer-exchanged into TP8 buffer (50 mM potassium acetate, 20 mM Tris acetate, pH 7.9) for free nucleotide incorporation assays or into TdT pH 6.5 storage buffer (200 mM KH_2_PO_4_, 100 mM NaCl, pH 6.5) for the generation of polymerase-nucleotide conjugates and were then concentrated to ~12 mg/mL using Vivaspin 20 columns (MWCO 30 kDa, Sartorius; Bohemia, NY, USA). Protein concentrations were determined by absorbance spectrophotometry on a NanoDrop 2000, assuming an extinction coefficient of 108,750 M^−1^ cm^−1^ at 280 nm. Protein aliquots were snap-frozen in liquid nitrogen and stored at −80 °C.

### 2.3. Determination of Enzyme Melting Temperatures

Enzyme melting temperatures were determined using a thermal shift assay with SYPRO Orange [13]. 10 μL samples containing 5 μM enzyme in TP8 buffer and 4x SYPRO Orange Protein Stain (Invitrogen; Waltham, MA, USA) were heated in a Bio-Rad CFX96 qPCR cycler with an initial equilibration of 2 min at 25 °C. The temperature was then increased to 95 °C (temperature increment: 0.5 °C; incubation time between increments: 1 min). Fluorescence was measured at the end of each incubation time (fluorescence filter set: ROX). The melting point was defined as the minimum of the first derivative of the fluorescence intensity with respect to the temperature.

### 2.4. Extension of Oligonucleotides by Free 2′,3′-Dideoxyribonucleoside-5′-Triphosphates (ddNTPs) Using TdT Variants

Extension reactions with free nucleotides contained 100 nM 5′-FAM (6-Carboxyfluorescein) labelled oligonucleotides (IDT, Table S2), 1 mM of one type of ddNTP (2′,3′-dideoxynucleoside triphosphate, GE Healthcare; Chicago, IL, USA), TP8 buffer (50 mM potassium acetate, 20 mM Tris acetate, pH 7.9) supplemented with divalent ions (RBC: 10 mM Mg acetate, 0.25 mM CoCl_2_; RBC without Mg^2+^: 0.25 mM CoCl_2_; TP8C: 1 mM CoCl_2_), 0.1 mg/mL BSA (Sigma-Aldrich; St. Louis, MI, USA), 0.005% Triton X-100 (Sigma-Aldrich), and 15 nM TdT unless indicated otherwise. The *substrate mix* (oligonucleotides, divalent ions, BSA and ddNTPs in TP8 buffer) was separately prepared from the *enzyme mix* (enzymes in TP8 buffer with Triton X-100). Both mixtures were equilibrated at the indicated reaction temperature for 30 s before starting the reaction by adding an equal volume of the *substrate mix* to the *enzyme mix* and mixing thoroughly with the pipette. All reactions were performed on a heat block. Samples were taken after 20, 40, 60, and 120 s by quenching a 2 μL sample into 8 μL quenching solution (6.25 mM EDTA, 93.75% (*v*/*v*) Hi-Di formamide (Applied Biosystems; Waltham, MA, USA)), unless otherwise specified. The 2 μL quenched samples were diluted into 18 μL analytical solution (75% Hi-Di formamide, 0.3 μL GeneScan 600 LIZ dye size standard) and analyzed by capillary electrophoresis (see capillary electrophoresis and data analysis below). Experiments were performed in triplicate unless otherwise specified.

### 2.5. Preparation of TdT-dTTP Conjugates

Polymerase-nucleotide conjugates were generated by tethering 5-Propargylamino-2′-deoxyuridine-5′-triphosphate (pa-dUTP, TriLink Biotechnologies; San Diego, CA, USA) to the one surface-exposed cysteine of MTdTc302-evo using a photocleavable amine-to-thiol crosslinker (Broadpharm; San Diego, CA, USA; catalog number BP-23354). The scheme for the preparation of linker-nucleotides and TdT-dNTP conjugates is described in detail in Palluk et al. [5]. Briefly, pa-dUTP was coupled to the crosslinker and ~30 nanomoles of purified product were reacted with ~20 nanomoles of MTdTc302-evo (=~12 mg/mL by A280, 150 μL in total) in TdT pH 6.5 storage buffer (200 mM KH_2_PO_4_, 100 mM NaCl, pH 6.5) for 1 h at room temperature. TdT-dNTP conjugates were subsequently purified using amylose affinity chromatography, buffer-exchanged into TP8 buffer, and then concentrated to 4 mg/mL. Aliquots were snap-frozen in liquid nitrogen and stored at −80 °C. 

### 2.6. Extension of Oligonucleotides Using TdT-dTTP Conjugates

Extension reactions with polymerase-nucleotide conjugates were identical to free ddTTP incorporation reactions in all respects except that the ddTTP and TdT were replaced by 0.025 mg/mL (~0.28 μM) TdT-dTTP conjugates. After quenching, the linker was photolyzed by 30 min of irradiation on a Benchtop 2UV Transilluminator (supplier: UVP, LLC; Upland, CA, USA; wavelength: 365 nm; irradiance: ~5 mW/cm^2^) to release extended oligonucleotides from TdT. The propargylamino “scar” left on the nucleobase after photolysis caused single oligonucleotide species to elute as two peaks in our capillary electrophoresis assay (see Palluk et al. [5]), so quenched samples were acetylated prior to electrophoresis to facilitate quantitation. Acetylation reactions contained equal volumes of quenched samples and acetylation solution (10 mM NHS acetate, 50 mM NaHCO_3_) and were incubated for 30 min at room temperature. Then, 2 μL of crude acetylation products were diluted into 18 μL analytical solution and analyzed by capillary electrophoresis. Experiments were conducted in triplicates, unless otherwise specified.

### 2.7. Capillary Electrophoresis and Data Analysis

Samples of 20 μL, containing 0.4 nM (incorporation of free nucleotides) or 2 nM (incorporation of conjugated nucleotides) 5′-FAM-labelled oligonucleotides and ~0.3 μL GeneScan 600 LIZ dye size standard in 75% Hi-Di formamide, were submitted to the UC Berkeley Sequencing Facility for capillary electrophoresis (CE). CE samples were run on an Applied Biosystems 3730xl DNA Analyzer with a 50 cm capillary array, containing POP-7 Polymer, with 15-s injection at 1.5 kV and a 41-min run at 15 kV, oven temperature of 68 °C, and buffer temperature of 35 °C. Electropherogram data files were processed using custom software written in R (r-project.org) with comparable functionality to the Peak Scanner software from Applied Biosystems. Substrate and product peak heights were measured to calculate relative yields at each time point of a time course. Each time course was fit to a monoexponential form in R (Formula: 1-*y* ~ exp(-k*x), where *y* = relative yield, *x* = time, and *k* = rate constant) and data are reported as the mean and standard deviation of the fitted rates for each set of replicates.

## 3. Results

### 3.1. Formation of 3′ Terminal DNA Structures Inhibits Substrate Elongation by TdT

**Figure 1:**
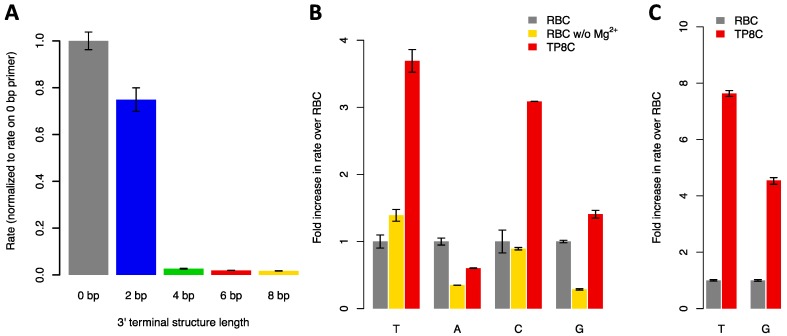
(**A**) Elongation of DNA primers with 3′ terminal hairpins of varying length with ddTTP using MTdTwt in RBC reaction buffer (50 mM potassium acetate, 20 mM Tris acetate, 10 mM magnesium acetate, 0.25 mM cobalt chloride, pH 7.9) at 37 °C (*n* = 3 replicates). Elongation rates were normalized to rates on the unstructured substrate (0 bp). (**B**) Elongation of an unstructured primer (P1) with ddNTPs using MTdTwt in reaction buffer (50 mM potassium acetate, 20 mM Tris acetate, pH 7.9) with varying M^2+^ concentrations: RBC (10 mM Mg^2+^, 0.25 mM Co^2+^), RBC without Mg^2+^ (0.25 mM Co^2+^), and TP8C (1 mM Co^2+^) (*n* = 2 replicates). Elongation rates were normalized to rates in RBC. (**C**) Elongation of an 8 bp hairpin primer (P5) with ddTTP or ddGTP in RBC and TP8C using MTdTwt (*n* = 3 replicates). Elongation rates were normalized to rates in RBC. In all of the above, error bars correspond to mean ± SD; T = ddTTP; A = ddATP; C = ddCTP; G = ddGTP.

Since it has been observed that TdT has reduced activity on double-stranded DNA substrates [9,14], we first sought to measure the elongation rates of primers with 3′ terminal guanine-cytosine (GC) hairpins of various lengths using murine TdT, expressed as MBP-fusion protein (herein, “MTdTwt”), to establish a quantitative baseline for improvements. To ensure that we were measuring the rate of the first nucleotide addition to the primer, we used 2′,3′-dideoxynucleotides (ddNTPs) that terminate elongation because they lack a 3′ hydroxy group (3′ OH). We observed decreased ddTTP (2′,3′-dideoxythymidine triphosphate) incorporation rates with increasing hairpin length, with the longest hairpin (8 bp) displaying a 57-fold slowdown compared to an unstructured poly-thymidine primer (Figure 1A, Figure S1).

### 3.2. Substitution of Mg^2+^ Cofactor with Co^2+^ Improves TdT Activity on Hairpin Substrates

The dependence of TdT on divalent metal ions (M^2+^) and the two-metal ion mechanism of nucleotide incorporation have been studied in detail [12,15,16,17,18] and it has been observed that Co^2+^ increases the activity of TdT on blunt-ended, double-stranded DNA substrates [19]. Our standard reaction buffer (RBC), which was identical to the TdT reaction buffer supplied by New England Biolabs, contained 10 mM Mg^2+^ and 0.25 mM Co^2+^, but TdT can use either cation as its exclusive cofactor [20]. Since divalent cations are known to stabilize duplex DNA [21], we hypothesized that removing the 10 mM Mg^2+^ from the reaction would lead to increased “fraying” of the hairpin and, thus, greater accessibility of its terminus to TdT, increasing the reaction rate [22]. However, since divalent cations are known to affect the base preference of TdT [20], we first measured the impact of removing Mg^2+^ on TdT-mediated elongation of an unstructured poly-thymidine primer (P1). Simply removing Mg^2+^ from the reaction significantly reduced the incorporation rates of the purine nucleotides ddATP (by 2.9x) and ddGTP (by 3.5x) but not of the pyrimidine nucleotides (Figure 1B, Figure S2), consistent with previous observations [20]. We hypothesized that the Co^2+^ concentration (0.25 mM) was sub-saturating, so we repeated the experiment with 1 mM Co^2+^. Under these conditions (buffer TP8C), the elongation rate of P1 by ddTTP, ddCTP, and ddGTP was 3.7-, 3.1-, and 1.4-times faster than in RBC, respectively, whereas the incorporation rate of ddATP was reduced by 40%. We then measured the elongation rate of the 8 bp hairpin primer P5 in TP8C to test whether this new condition would increase the elongation rate of a hairpin. Compared to RBC, P5 was elongated 7.9x faster in TP8C by ddTTP and 4.5x faster by ddGTP. Notably, these improvements were ~2x greater than the improvements seen with P1, suggesting that the change in divalent cation condition disproportionally improved the activity of TdT on structured substrates (Figure 1C, Figure S3).

### 3.3. Computational Protein Design Yields a TdT Variant with Enhanced Thermostability

DNA secondary structures can be melted by raising the temperature, suggesting that elongation rates of hairpins by TdT would be improved by elevating the reaction temperature. However, murine TdT is heat-sensitive [23], so the wild-type enzyme is unsuitable for this purpose. In order to generate a TdT variant that could operate at elevated temperatures, we used the FireProt web server [11] to suggest 15 point mutations that are predicted to enhance enzyme thermostability [10] (Table S3). We expressed and purified the combined mutant (MTdT-evo) as an MBP-fusion protein (Figure S8) and found that its melting temperature was 50 °C, an improvement of 8.8 °C over MTdTwt (Figure S4). Unlike MTdTwt, MTdT-evo had full activity at 47 °C and the elongation rate of P1 by MTdT-evo was approximately the same at 37 and 47 °C (Figure S5).

### 3.4. Elevated Reaction Temperature and Optimized Divalent Ion Concentrations Synergistically Accelerate Incorporation of Free and Conjugated Nucleotides into a Hairpin Primer

**Figure 2:**
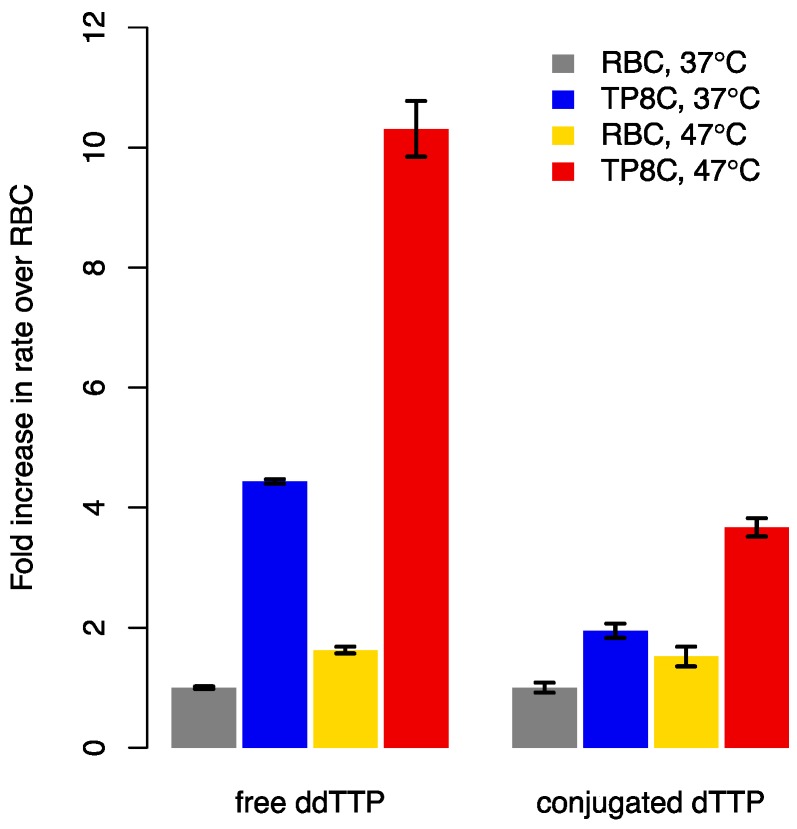
Optimizing the divalent cation concentrations and elevating the reaction temperature by 10 °C synergistically increased the incorporation rates of free and conjugated nucleotides into a hairpin primer. Elongation of hairpin primer P5 by either free ddTTP and MTdT-evo or MTdTc302-evo-dTTP conjugates were performed in RBC and TP8C buffer at 37 °C and 47 °C. Elongation rates were normalized to rates in RBC at 37 °C. Error bars correspond to mean ± SD of *n* = 3 independent replicates.

Next, we investigated the effect of an elevated reaction temperature on the elongation rate of hairpin primer P5 by ddTTP. At 47 °C, MTdT-evo extended P5 2.3x faster than at 37 °C in TP8C, whereas the elongation rate of P1 was similar at both temperatures (Figure S7). By contrast, performing the reaction in RBC only led to a 1.6x rate enhancement with the temperature increase, suggesting a synergistic effect between the divalent cation and temperature improvements (Figure 2, Figure S6). Combining these improvements, we achieved a 10.3x enhancement in the elongation rate of P5 by ddTTP compared to our initial conditions. Finally, we examined whether our findings also applied to the elongation of oligonucleotides using polymerase-nucleotide conjugates [5]. To prepare conjugates from MTdT-evo, we first had to remove all surface-accessible cysteine residues except for one to enable single site-specific attachment of linker-dTTP. The single-surface-cysteine variant, MTdTc302-evo, had a slightly reduced melting temperature (48.5 °C, Figure S4) compared to MTdT-evo, suggesting that the cysteine mutations were subtly destabilizing, but the variant had broadly similar properties to MTdT-evo (Figure S5). We then generated MTdTc302-evo-dTTP conjugates and measured the elongation rate of hairpin primer P5 by conjugated dTTP. At 47 °C, the conjugates elongated P5 1.9x faster than at 37 °C in TP8C (and 1.5x faster in RBC) for a combined improvement of 3.7x over our initial conditions (Figure 2, Figure S6).

## 4. Discussion

Here, we demonstrated that wild-type TdT substantially reduced activity on primers with 3′ terminal structure and we described two synergistic approaches for improving its activity on these substrates: Optimizing the divalent ion concentrations and elevating the reaction temperature. The latter required engineering TdT for enhanced thermostability. By combining these approaches, we achieved a ~10-fold increase in the rate elongation of a GC hairpin primer by ddTTP. We then implemented these improvements in our recently described DNA synthesis method based on TdT-dNTP conjugates [5] and found a ~4-fold improvement in the elongation rate of the same hairpin. 

Both approaches were inspired by conditions known to reduce the stability of base pairing [22]. TdT binds to the last four nucleotides of a ssDNA substrate [12] and excludes dsDNA substrates from entering its catalytic site due its unique *Loop1* [24]. An appealing explanation for the observed rate enhancements is that the optimized conditions lead to a population increase of the “frayed” state of the hairpin wherein the last 4 bases are free to enter the TdT catalytic site. However, further experiments are needed to definitively attribute the rate enhancement to reduced stability or enhanced dynamics of the terminal structures. A deeper exploration of divalent cation concentrations and mixtures may yet reveal superior conditions to TP8C. Although we did not explore the effect of varying the monovalent cation concentrations in this study, we view this as a potentially fruitful area for future work since monovalent cations are also known to affect the stability and dynamics of DNA structures [25]. Finally, we note that our results do not rule out the possibility that adjusting the divalent ion concentrations could affect the activity of TdT on hairpins by other means.

This study represents a first step towards improving TdT activity on structured substrates, addressing a critical hurdle in the development of enzymatic oligonucleotide synthesis methods. While we achieved a ~10-fold increase in the elongation rate of a hairpin primer under our optimized conditions compared to the standard conditions, this rate was still ~6-fold slower than the elongation rate of an unstructured primer in the standard conditions. As such, greater optimization is required to access TdT’s full activity on structured substrates. We speculate that elevating the temperature further will sufficiently “fray” any hairpin to the point where it behaves like an unstructured primer, but testing this hypothesis will require further engineering of TdT for thermostability.

An alternative and potentially complementary approach to enhance the activity of TdT on hairpin substrates is to supplement the reactions with PCR additives, such as (dilute) DMSO or betaine, that tend to destabilize DNA structures. TdT engineering would likely be required to enable activity in the presence of effective concentrations of these additives.

Although we did not achieve parity between the elongation rates of structured and unstructured primers, we believe that simultaneously optimizing the cation concentrations and elevating the reaction temperature represents a promising approach for further enhancing the activity of TdT on structured substrates. Eliminating the inhibitory effects of DNA secondary structures is crucial to enable consistent reaction times and the extremely high stepwise yields required to exceed the performance of the phosphoramidite method and unlock the full potential of enzymatic oligonucleotide synthesis for emerging synthetic biology and “digital biology” applications.

## Supplementary Materials

The following are available online at https://www.mdpi.com/2073-4425/11/1/102/s1, Figure S1: Time course of elongation of oligonucleotides with 3′ terminal hairpins of varying lengths (P1–P5, Table S2) with ddTTP using MTdTwt; Figure S2: Elongation rates of an unstructured primer by MTdTwt vary by nucleobase and depend on the divalent ion concentrations; Figure S3: Time course data of the elongation of a 3′ terminal 8 bp hairpin primer (P5) with ddTTP (T) or ddGTP (G) in RBC or TP8C buffer using MTdTwt at 37 °C; Figure S4: Engineered TdT mutants MTdT-evo and MTdTc302-evo display enhanced thermostability; Figure S5: Thermostability engineering of TdT enables full activity at 47 °C; Figure S6: Time courses of elongation of a 3′ terminal 8 bp hairpin primer (P5) with free and conjugated nucleotides under optimized divalent cation conditions and at elevated temperature; Figure S7: Raising the reaction temperature by 10°C enhances the elongation rate of hairpin primer P5 (8 bp) but not of unstructured primer P1 (0 bp).; Figure S8: SDS-PAGE analysis of eluted MTdTwt and MTdT-evo after immobilized metal ion affinity chromatography (IMAC); Table S1: Plasmid and strain accession numbers; Table S2: List of oligonucleotides used in enzyme activity assays; Table S3: Overview of suggested mutations in MTdTwt by the FireProt algorithm to enhance thermostability; Note S1: Protein sequences of MBP-TdT fusion proteins used within this study [26,27].
