# Supplementary material for: Enhancing Terminal Deoxynucleotidyl Transferase Activity on Substrates with 3′ Terminal Structures for Enzymatic De Novo DNA Synthesis
Source: Genes (Basel). 2020 Jan 16;11(1):102. doi: 10.3390/genes11010102 (PMC7016565; doi:10.3390/genes11010102)
Supplement: Supplementary file 1 [file genes-11-00102-s001.pdf]

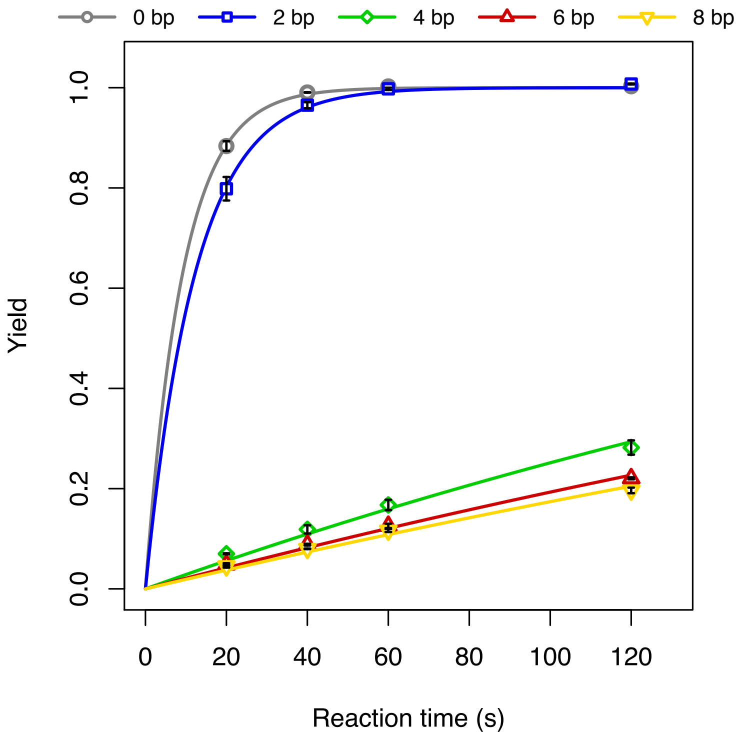

Supplemental Figure S1.

Time course of elongation of oligonucleotides with 3' terminal hairpins of varying lengths (P1 – P5, Table S2) with ddTTP using MTdTwt. Reactions were performed in RBC reaction buffer at 37 °C. Samples were quenched after 20 s, 40 s, 60 s, and 120 s, and were analyzed by capillary electrophoresis. Data are mean ± SD of *n = 3* independent replicates. Curves represent monoexponential fits to the time courses; rate estimates are summarized in Figure 1A.

A

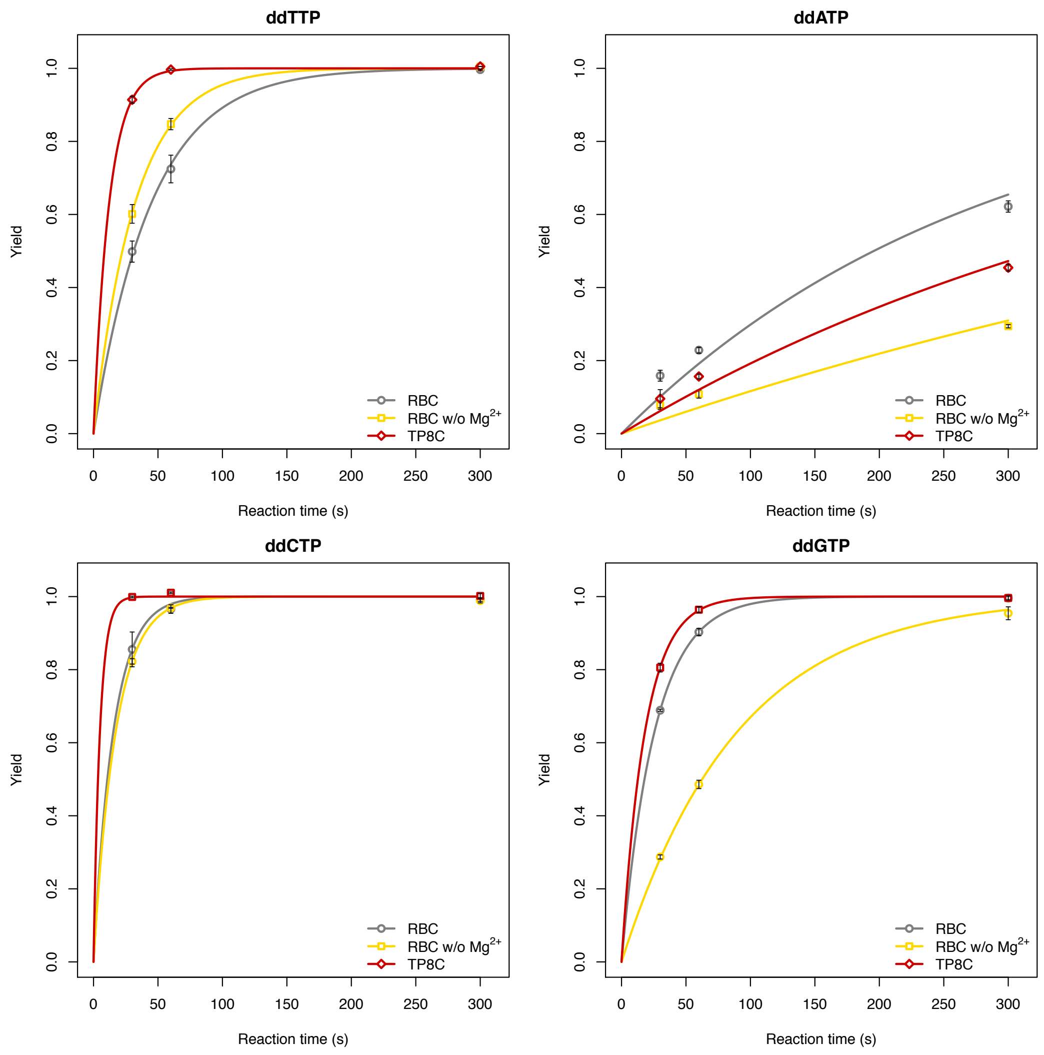

B

**2',3'-dideoxynucleotide**	**Rate x 10^3 [s^{-1}]**		
	**RBC**	**RBC w/o Mg^{2+}**	**TP8C**
**ddTTP**	22	31	82
**ddATP**	3.5	1.2	2.1
**ddCTP**	65	58	200
**ddGTP**	39	11	55

Supplemental Figure S2.

Elongation rates of an unstructured primer by MTdTwt vary by nucleobase and depend on the divalent ion concentrations. (**A**) Time course of elongation of an unstructured primer (P1) with each ddNTP using 5 nM MTdTwt. Reactions were performed at 37 °C, and samples were quenched after 20 s, 60 s, and 300 s, and were analyzed by capillary electrophoresis. Data are mean ± SD of $n = 2$ independent replicates. Curves represent monoexponential fits to the time courses. (**B**) Table of rate estimates from the fits shown in Figure S2A. Relative rates (normalized to the rate in RBC) are summarized in Figure 1B.

Supplemental Figure S3

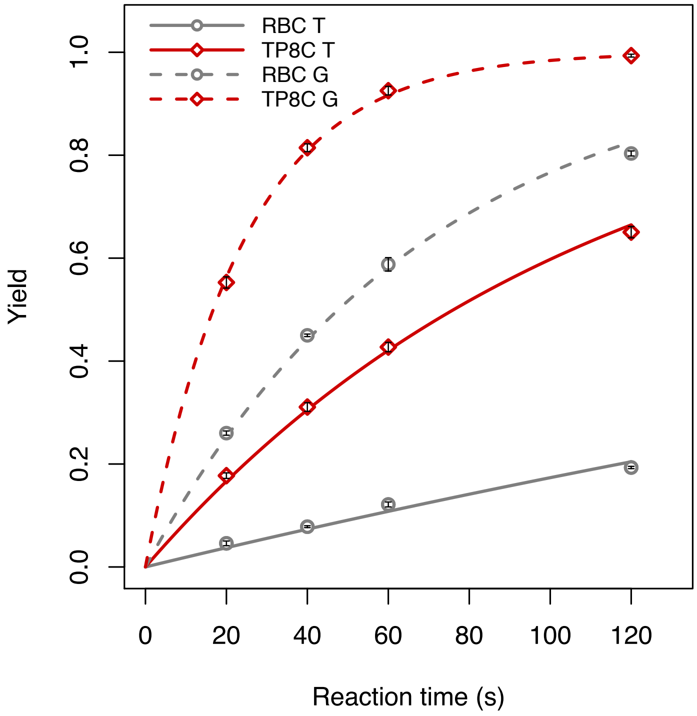

Supplemental Figure S3.

Time course data of the elongation of a 3' terminal 8 bp hairpin primer (P5) with ddTTP (T) or ddGTP (G) in RBC or TP8C buffer using MTdTwt at 37 °C. Samples were quenched after 20 s, 40 s, 60 s, and 120 s, and analyzed by capillary electrophoresis. Data are mean ± SD of *n = 2* independent replicates. Curves represent monoexponential fits to the time courses; rate estimates are summarized in Figure 1C.

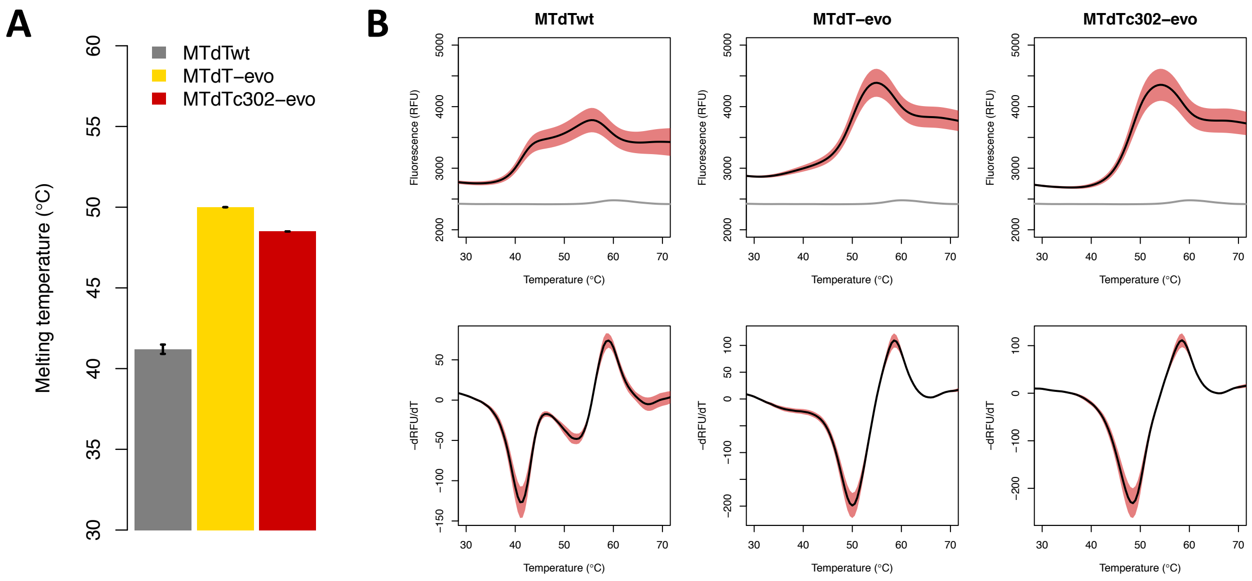

Supplemental Figure S4.

Engineered TdT mutants MTdT-evo and MTdTc302-evo display enhanced thermostability. (**A**) Estimated melting temperatures of MTdTwt, MTdT-evo and MTdTc302-evo (41.2 °C, 50.0 °C, and 48.5 °C, respectively) determined using a thermal shift assay. (**B**) Thermal shift assay melt curves: SYPRO Orange fluorescence measure during a temperature ramp of the respective proteins, (upper row) and first derivative (lower row). The melting temperature is estimated by the temperature at which the slope of the fluorescence reaches a minimum value. Data are mean ± SD of n = 3 independent replicates.

A

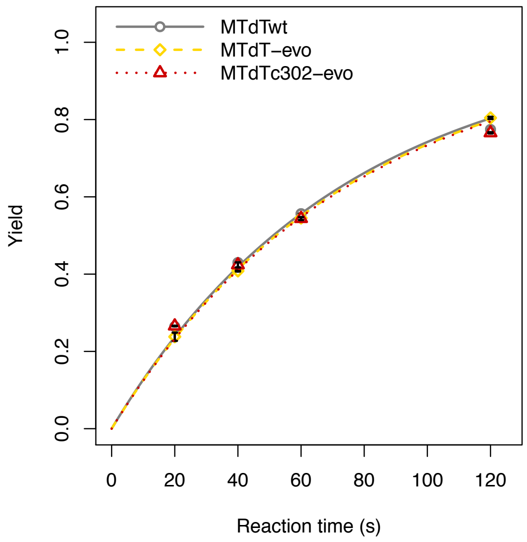

B

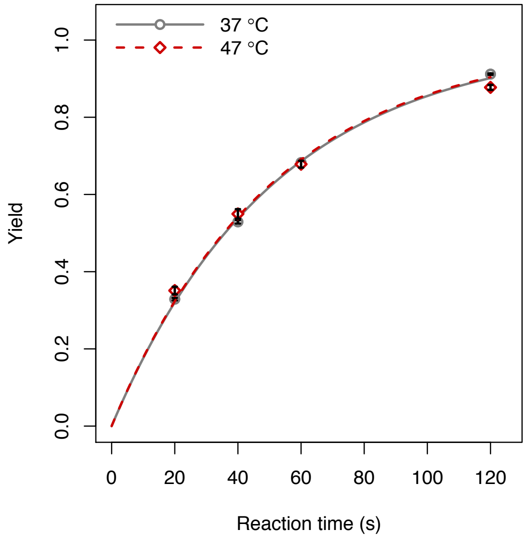

Supplemental Figure S5.

Thermostability engineering of TdT enables full activity at 47 °C. (**A**) Time course of elongation of unstructured primer P1 by 5 nM MTdTwt, MTdT-evo and MTdTc302-evo with ddTTP at 37 °C. (**B**) Time course of elongation of unstructured primer P1 by 5 nM MTdT-evo with ddTTP at 37 °C and 47 °C. Reactions were performed in RBC, and samples were quenched after 20 s, 40 s, 60 s, and 120 s, and analyzed by capillary electrophoresis. Data are mean ± SD; *n = 2* independent replicates. Curves represent monoexponential fits to the time courses.

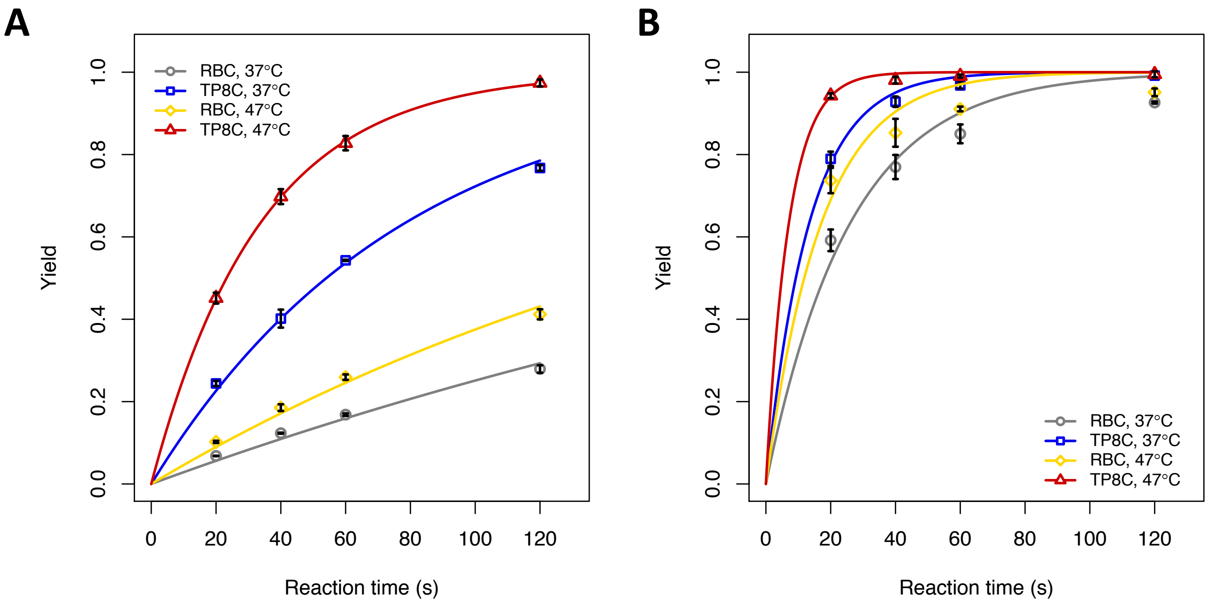

Supplemental Figure S6.

Time courses of elongation of a 3’ terminal 8 bp hairpin primer (P5) with free and conjugated nucleotides under optimized divalent cation conditions and at elevated temperature. Reactions were performed in RBC or TP8C at 37 °C or 47 °C using MTdT-evo and ddTTP (**A**) or MTdTc302-evo-dTTP conjugates (**B**). Samples were taken after 20 s, 40 s, 60 s and 120 s, and analyzed by capillary electrophoresis. Data are mean ± SD; *n = 3* independent replicates. Curves represent monoexponential fits to the time courses; rate estimates are summarized in Figure 2.

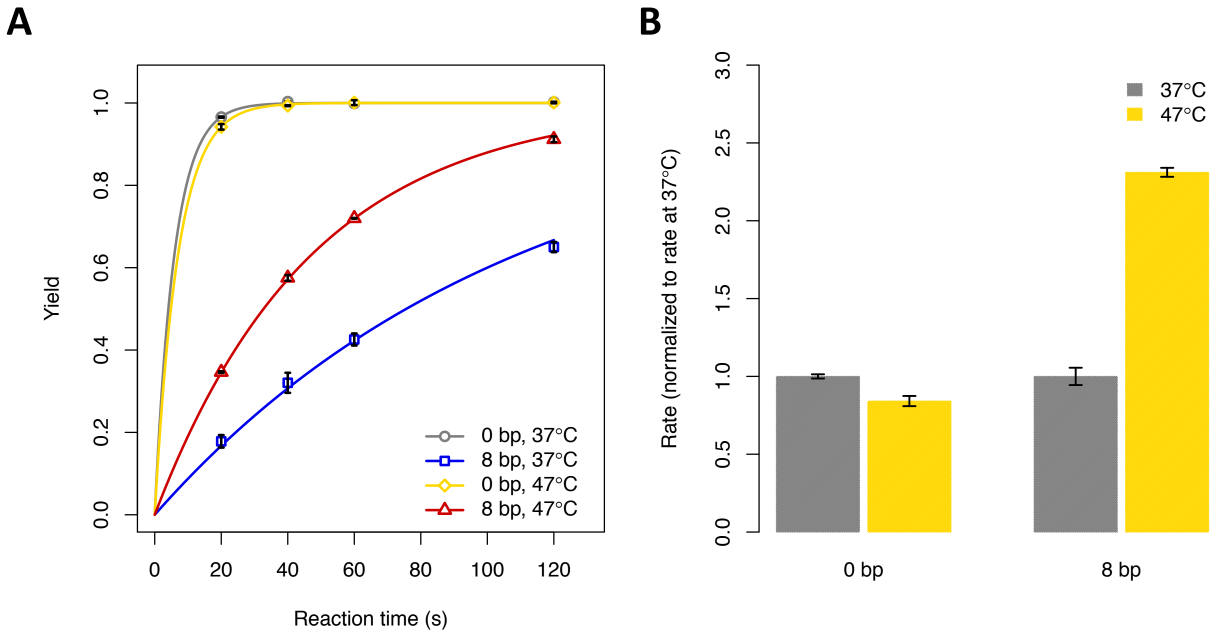

Supplemental Figure S7.

Raising the reaction temperature by 10°C enhances the elongation rate of hairpin primer P5 (8 bp) but not of unstructured primer P1 (0 bp). Time courses (**A**), and normalized rate estimates (**B**) of reactions were performed in TP8C with free ddTTP using 15 nM MTdT-evo at 37 °C or 47 °C. Samples were taken after 20 s, 40 s, 60 s and 120 s, and analyzed by capillary electrophoresis. Data are mean ± SD; *n = 2* independent replicates. Curves represent monoexponential fits to the time courses; rate estimates were normalized to estimates at 37 °C.

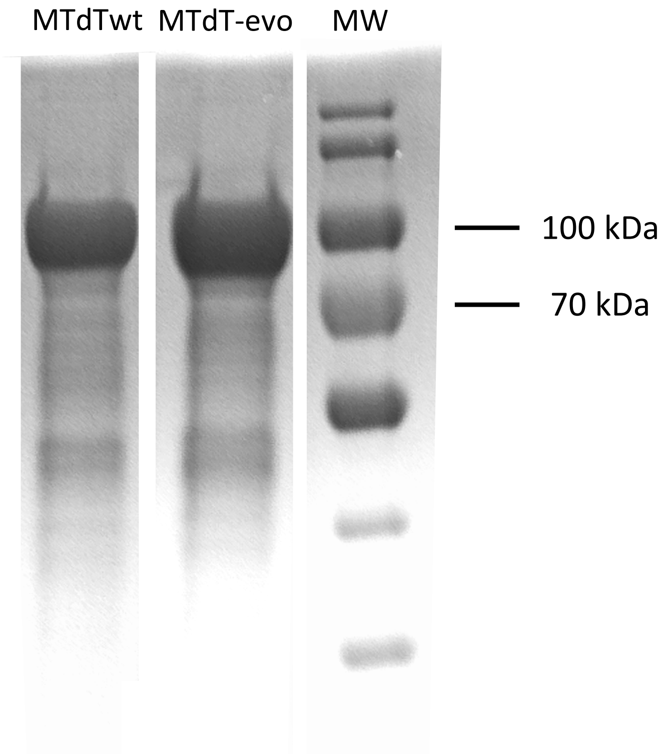

Supplemental Figure S8.

SDS-PAGE analysis of eluted MTdTwt and MTdT-evo after immobilized metal ion affinity chromatography (IMAC). Enzymes were expressed in *E. coli* BL21 (DE3) and purified using gravity columns loaded with Ni-NTA agarose resin. The procedure is described in the methods section of the main text. 5 µL elution were loaded on an 8-16% polyacrylamide gel (BioRad). Gels were stained with SafeStain (Thermo Scientific). MW = molecular weight marker.

Supplemental Table S1.

Plasmid and strain accession numbers. Sequences of the plasmids coding for all TdT variants can be downloaded from the JBEI Public registry (https://public-registry.jbei.org/folders/432). Respective expression strains harboring the plasmids were added to the JBEI strain archive and are available upon request.

Construct	Plasmid	Expression Strain
pET19b-MTdTwt	JPUB_010253	JPUB_010269
pET19b-MTdT-evo	JPUB_013230	JPUB_013229
pET19b-MTdTc302-evo	JPUB_013228	JPUB_013227

Supplemental Table S2.

List of oligonucleotides used in enzyme activity assays. All primers were purchased HPLC purified from Integrated DNA Technologies (IDT). Melting temperatures (T_m) were calculated using the mFold algorithm [1] with ion concentration of the RBC buffer (20 mM Tris acetate, 50 mM Potassium acetate, 10 mM Magnesium acetate, 0.25 mM Cobalt(II) chloride, pH 7.9).

**Name**	**Sequence (5' - - > 3')**	**Note**
P1	/56-FAM/TTTTTTTTTTTTTTTTTTTTTTTTTTTTTTTTTTTTTTTTTTTTTTTTTTTTTTTTTTTT	5' fluorescein;<br>T homopolymer;<br>T_m = /
P2	/56-FAM/TTTTTTTTTTTTTTTTTTTTTTTTTTTTTTTTTTTTTTTTTTTTTTTTTTTTTGCTTTGC	5' fluorescein;<br>3' 2 bp GC hairpin;<br>T_m = 20.9 °C
P3	/56-FAM/TTTTTTTTTTTTTTTTTTTTTTTTTTTTTTTTTTTTTTTTTTTTTTTTTGCGGTTTCCGC	5' fluorescein;<br>3' 4 bp GC hairpin;<br>T_m = 67.7 °C
P4	/56-FAM/TTTTTTTTTTTTTTTTTTTTTTTTTTTTTTTTTTTTTTTTTTTTTGCGGCGTTTCGCCGC	5' fluorescein;<br>3' 6 bp GC hairpin;<br>T_m = 85.0 °C
P5	/56-FAM/TTTTTTTTTTTTTTTTTTTTTTTTTTTTTTTTTTTTTTTTTGCGGCGCGTTTCGCGCCGC	5' fluorescein;<br>3' 8 bp GC hairpin;<br>T_m = 93.4 °C

Supplemental Table S3

Overview of suggested mutations in MTdTwt by the FireProt algorithm to enhance thermostability. Mutations are picked from multiple sequence alignments either by majority (> 50%) or ratio (> 40% and 5x frequency of wildtype residue) in this position. Point mutants are created *in silico* and the change in folding energy due to the mutation ($\Delta\Delta G$) is predicted by FoldX [2, 3].

Total $\Delta\Delta G$ = -3.65 kcal/mol (16 mutations)			
**Mutation**	**Prevalent by majority**	**Prevalent by ratio**	**$\Delta\Delta G$ [kcal/mol]**
D173E	Y	Y	0.40
D179A	Y	Y	-1.20
L181F	Y	Y	-0.29
S187R	Y	N	-0.49
S195A	Y	Y	-1.16
T211L	Y	N	-1.95
I214L	Y	N	-0.09
S223R	N	Y	-0.10
G227E	Y	Y	-0.11
I229L	Y	N	-0.57
F267Y	Y	N	0.30
F285L	Y	N	0.44
D325G	Y	N	0.28
M330L	Y	N	0.06
T354G	Y	N	-0.29
L398M*	Y	N	-0.15

* = not implemented in MTdT-evo and MTdTc302-evo

Supplemental Note S1

Protein sequences of MBP-TdT fusion proteins used within this study.

>MTdTwt (10xHis-MBP-TdT; wildtype)

MGHHHHHHHHHHSSGHIDDDDKHMMKIEEGKLVIWINGDKGYNGLAEVGKKFEKDTGIKVTVEHPDKLEEKFPQVAATGDGPDIIFWAHDRFG
GYAQSGLLAEITPDKAFQDKLYPFTWDAVRYNGKLIAYPIAVEALSLIYNKDLLPNPPKTWEEIPALDKELKAKGKSALMFNLQEPYFTWPLIAADGG
YAFKYENGKYDIKDVGVDNAGAKAGLTFLVDLIKNKHMNADTDYSIAEAAFNKGETAMTINGPWAWSNIDTSKVNYGVTVLPTFKGQPSKPFVGV
LSAGINAASPNKELAKEFLENYLLTDEGLEAVNKDKPLGAVALKSYEEELVKDPRIAATMENAQKGEIMPNIPQMSAFWYAVRTAVINAASGRQTV
DEALKDAQTNSSSNNNNNNNNNNLGIEGRISHMSMGGRDIVDGSEFSPSPVPGSQNVPAPAVKKISQYACQRRTTLNNYNQLFTDALDILAENDE
LRENEGSCLAFMRASSVLKSLPFPITSMKDTEGIPCLGDKVKSIIEGIIEDGESSEAKAVLNDERYKSFKLFTSVFGVGLKTAEKWFRMGFRTLSKIQSDK
SLRFTQMQKAGFLYYEDLVSCVNRPEAEAVSMLVKEAVVTFLPDALVTMTGGFRRGKMTGHDVDFLITSPEATEDEEQQLLHKVTDFWKQQGLLL
YCDILESTFEKFKQPSRKVDALDHFQKCFLILKLDHGRVHSEKSGQQEGKGWKAIRVDLVMCPYDRRAFALLGWTGSRQFERDLRRYATHERKMM
LDNHALYDRTKRVFLEAESEEEIFAHLGLDYIEPWERNA

>MTdT-evo (10xHis-MBP-TdT; multipoint mutant suggested by the FireProt algorithm [2])

MGHHHHHHHHHHSSGHIDDDDKHMMKIEEGKLVIWINGDKGYNGLAEVGKKFEKDTGIKVTVEHPDKLEEKFPQVAATGDGPDIIFWAHDRFG
GYAQSGLLAEITPDKAFQDKLYPFTWDAVRYNGKLIAYPIAVEALSLIYNKDLLPNPPKTWEEIPALDKELKAKGKSALMFNLQEPYFTWPLIAADGG
YAFKYENGKYDIKDVGVDNAGAKAGLTFLVDLIKNKHMNADTDYSIAEAAFNKGETAMTINGPWAWSNIDTSKVNYGVTVLPTFKGQPSKPFVGV
LSAGINAASPNKELAKEFLENYLLTDEGLEAVNKDKPLGAVALKSYEEELVKDPRIAATMENAQKGEIMPNIPQMSAFWYAVRTAVINAASGRQTV
DEALKDAQTNSSSNNNNNNNNNNLGIEGRISHMSMGGRDIVDGSEFSPSPVPGSQNVPAPAVKKISQYACQRRTTLNNYNQLFTDALEILAENAE
FRENEGRCLAFMRAASVLKSLPFPITSMKDLEGLPCLGDKVKRIIEEILEDGESSEAKAVLNDERYKSFKLFTSVFGVGLKTAEKWYRMGFRTLSKIQSD
KSLRLTQMQKAGFLYYEDLVSCVNRPEAEAVSMLVKEAVVTFLPGALVTLTGGFRRGKMTGHDVDFLITSPEAGEDEEQQLLHKVTDFWKQQGLL
LYCDILESTFEKFKQPSRKVDALDHFQKCFLILKLDHGRVHSEKSGQQEGKGWKAIRVDLVMCPYDRRAFALLGWTGSRQFERDLRRYATHERKM
MLDNHALYDRTKRVFLEAESEEEIFAHLGLDYIEPWERNA

>MTdTc302-evo (10xHis-MBP-TdT; multipoint mutant suggested by the FireProt algorithm [2], single attachment site: Cys302)

MGHHHHHHHHHHSSGHIDDDDKHMMKIEEGKLVIWINGDKGYNGLAEVGKKFEKDTGIKVTVEHPDKLEEKFPQVAATGDGPDIIFWAHDRFG
GYAQSGLLAEITPDKAFQDKLYPFTWDAVRYNGKLIAYPIAVEALSLIYNKDLLPNPPKTWEEIPALDKELKAKGKSALMFNLQEPYFTWPLIAADGG
YAFKYENGKYDIKDVGVDNAGAKAGLTFLVDLIKNKHMNADTDYSIAEAAFNKGETAMTINGPWAWSNIDTSKVNYGVTVLPTFKGQPSKPFVGV
LSAGINAASPNKELAKEFLENYLLTDEGLEAVNKDKPLGAVALKSYEEELVKDPRIAATMENAQKGEIMPNIPQMSAFWYAVRTAVINAASGRQTV
DEALKDAQTNSSSNNNNNNNNNNLGIEGRISHMSMGGRDIVDGSEFSPSPVPGSQNVPAPAVKKISQYACQRRTTLNNYNQLFTDALEILAENAE
FRENEGRALAFMRAASVLKSLPFPITSMKDLEGLPSLGDKVKRIIEEILEDGESSEAKAVLNDERYKSFKLFTSVFGVGLKTAEKWYRMGFRTLSKIQSD
KSLRLTQMQKAGFLYYEDLVSCVNRPEAEAVSMLVKEAVVTFLPGALVTLTGGFRRGKMTGHDVDFLITSPEAGEDEEQQLLHKVTDFWKQQGLL
LYADILESTFEKFKQPSRKVDALDHFQKCFLILKLDHGRVHSEKSGQQEGKGWKAIRVDLVMSPYDRRAFALLGWTGSRQFERDLRRYATHERKM
MLDNHALYDRTKRVFLEAESEEEIFAHLGLDYIEPWERNA*

Supplemental References

1. Zuker, M. Mfold web server for nucleic acid folding and hybridization prediction. *Nucleic Acids Research* **2003**, *31*, 3406-3415, doi:10.1093/nar/gkg595.
2. Bednar, D.; Beerens, K.; Sebestova, E.; Bendl, J.; Khare, S.; Chaloupkova, R.; Prokop, Z.; Brezovsky, J.; Baker, D.; Damborsky, J. FireProt: Energy- and Evolution-Based Computational Design of Thermostable Multiple-Point Mutants. *PLOS Computational Biology* **2015**, *11*, e1004556, doi:10.1371/journal.pcbi.1004556.
3. Schymkowitz, J.; Borg, J.; Stricher, F.; Nys, R.; Rousseau, F.; Serrano, L. The FoldX web server: an online force field. *Nucleic Acids Research* **2005**, *33*, W382-W388, doi:10.1093/nar/gki387.
